# Computational Insight into the Effect of Natural Compounds on the Destabilization of Preformed Amyloid-β(1–40) Fibrils

**DOI:** 10.3390/molecules23061320

**Published:** 2018-05-31

**Authors:** Francesco Tavanti, Alfonso Pedone, Maria Cristina Menziani

**Affiliations:** Department of Chemical and Geological Sciences, University of Modena and Reggio Emilia, Via G. Campi 103, 41125 Modena, Italy; alfonso.pedone@unimore.it

**Keywords:** molecular dynamics simulation, biophenols, natural compounds, amyloid fibrils, Alzheimer’s disease, ligand–protofiber interactions

## Abstract

One of the principal hallmarks of Alzheimer’s disease (AD) is related to the aggregation of amyloid-β fibrils in an insoluble form in the brain, also known as amyloidosis. Therefore, a prominent therapeutic strategy against AD consists of either blocking the amyloid aggregation and/or destroying the already formed aggregates. Natural products have shown significant therapeutic potential as amyloid inhibitors from in vitro studies as well as in vivo animal tests. In this study, the interaction of five natural biophenols (curcumin, dopamine, (-)-epigallocatechin-3-gallate, quercetin, and rosmarinic acid) with amyloid-β(1–40) fibrils has been studied through computational simulations. The results allowed the identification and characterization of the different binding modalities of each compounds and their consequences on fibril dynamics and aggregation. It emerges that the lateral aggregation of the fibrils is strongly influenced by the intercalation of the ligands, which modulates the double-layered structure stability.

## 1. Introduction

The pathological hallmark of Alzheimer’s disease (AD) is the extracellular accumulation of insoluble proteinaceous deposits called amyloid fibrils [1] that induce cytotoxicity. The formation of mature amyloid fibrils (Aβ) proceeds through a nucleation-dependent process, where monomers and oligomers aggregate together, forming β-sheet-rich protein structures. The most common fibrils are Aβ(1–40) and Aβ(1–42), which are composed of 40 and 42 amino acids, respectively, and are characterized by β-strand units aligned perpendicularly to the main fibril axis [2]. Destabilization and clearance of amyloid aggregates by small molecules is one of the promising approaches towards the development of AD therapies [3].

In recent years, epidemiological studies on the effects of the diet against AD and dementia suggested that the high intake of flavonoids and polyphenols found in fruits and vegetables reduces the risk of AD and cognitive impairments, and several natural molecules have been identified as promoting cognitive health and interfering with the amyloidogenic activity in AD [4].

A detailed knowledge of how these molecules interact with Aβ fibrils is a prerequisite for the design of new efficient drugs. Unfortunately, despite intensive research, the experimental characterization of full-length Aβ oligomers/inhibitor complexes at a high level of resolution remains a great challenge.

Atomistic computer simulations are well-suited to provide molecular-level details of amyloid oligomer and fibril interactions with ligands, helping in the future development and characterization of druggable modalities [5]. Basically, four aspects of the flavonoid–amyloid interactions have been studied by computational methods: (1) the effect of ligands on the conformational transitions of Aβ monomers from an initial random coil or α-helix into β-sheet structures [6,7] and ligand-mediated conformational changes of the Aβ dimer [8] by means of replica exchange molecular dynamics (REMD) simulations; (2) the effect of ligands on the aggregation of Aβ(17–36) using coarse-grained simulations [9]; (3) the effect of ligands on the conformation and stability of amyloid-beta mutants [10] by molecular dynamics (MD) simulations; (4) the preferential binding sites of ligands and their effect on amyloid structure dynamics [11], Aβ fragments, and full-length single Aβ protofilaments [12,13,14,15,16,17,18] by means of docking experiments, MD simulations, and free energy calculations.

Although recently, a few studies devoted their attention to the interaction of ligands (mainly markers for amyloid detection [19,20,21]) with multiple Aβ protofilaments, to the best of our knowledge, this aspect has not been investigated thoroughly for natural polyphenol ligands, except for curcumin [12].

In this study, the binding modalities of five natural biophenols (curcumin, dopamine, (-)-epigallocatechin-3-gallate, quercetin, and rosmarinic acid) with single Aβ(1–40) protofilaments and double-layer oligomer aggregates will be studied through atomistic computational simulations, in order to explore structural changes in aggregate pathways upon binding.

First, putative binding sites on the Aβ(1–40) protofibril will be explored by replica exchange molecular dynamics (REMD) simulations. Then, binding free energies (ΔG_bind_) will be computed on the complexes to determine the thermodynamically favored binding modalities. Finally, the structural effects caused by the binding of polyphenols to two double-layer protofilament polymorphs will be assessed. To this goal, the determination of the stability of the sheet-to-sheet associations of the double-layered organizations with and without the polyphenols will be computed by means of the potential of mean force (PMF) methodology.

## 2. Methods

### 2.1. Molecular Dynamics Simulations

Molecular dynamics simulations were performed with GROMOS 54a7 force field [22]. The structural model of amyloid fibrils was retrieved from the Protein Data Bank [23] (PDB ID: 2LMN [24]). From this structure, an Aβ monomer was isolated and the missing N-terminal peptide region of the Aβ(1–40) monomer (^1^DAEFRHDS^8^) was built using the Molefacture plugin in the VMD package [25] as random coils as predicted by both the Jpred web server [26] and by the Modeller package [27] for protein secondary structure assignments. Standard protonation states corresponding to pH 7 were assigned to ionizable residues. The Aβ(1–40) protofibril was composed by repeating 10 monomeric units along its principal axis, obtaining a continuous structure 5 nm long.

The force field assigned to each ligand in their standard protonation states at pH 7 was built in the GROMACS format [28] by using the Automated Topology Builder [29,30] web server.

The simulation box (7.5 × 9.7 × 8.0 nm) contains one Aβ(1–40) protofibril composed by repeating 10 monomeric units, with one ligand placed in a random position with respect to the fibril, and about 30,000 simple point charge water molecules [31]. Counter ions (Na^+^ and Cl^−^) were added at random locations to neutralize the systems, with an ion concentration of 150 mM, close to the physiological value.

All the simulations were carried out at physiological temperature (310 K) and pressure of 1 bar. The systems were first equilibrated for 2 ns in the NVT ensemble, then 10 n runs were carried out in the NPT ensemble. The temperature was controlled using a velocity-rescaling thermostat with a coupling time of 0.1 ps. During equilibration, the Berendsen barostat was used to control the pressure, while during the production run, the Parrinello–Rhaman barostat was used with coupling time of 2 ps and an isothermal compressibility of 4.5 × 10^−5^ bar^−1^, and the timestep used was 2.0 fs. The particle-mesh Ewald algorithm was used to calculate long-range electrostatics [32], with a fourth-order cubic interpolation, a grid spacing of 0.16 nm, and a real-space cutoff of 1 nm [33]. Both Van der Waals and neighbor list cutoffs describing short-range interactions were set to 1.0 nm. A production run of 50 ns was used to identify the ligand binding sites (Section 2.2), whereas trajectories of 100 ns were necessary for the computation of the stability of the different protofibril polymorphs (Section 2.4). Data analysis was performed using the GROMACS-5.0.4 package [34].

### 2.2. Ligand Binding Sites

Temperature replica exchange MD (REMD) simulations were used to define the most probable interacting sites of each compound with the Aβ(1–40) protofibrils. The temperatures used for replicas were obtained by the work of Patriksson and van der Spoel [35] and are reported below: 300.00, 301.16, 302.32, 303.49, 304.66, 305.83, 307.01, 308.19, 309.38, 310.57, 311.76, 312.96, 314.16, 315.37, 316.57, 317.78, 319.00, 320.22, 321.44, 322.66, 323.89, 325.12, 326.36, 327.60, 328.85, 330.09, 331.34, 332.60, 333.86, 335.12, 336.39, 337.66, 338.93, 340.21.

An acceptance ratio of 20% was chosen, as previously suggested by Ngo et al. [36]. Each REMD simulation replica was equilibrated with an NVT and an NPT ensemble with the same parameters as for MD simulations. Then, a 50 ns run (i.e., the production run) was performed for each replica, and exchanges between neighboring replicas were checked every 500 steps corresponding to 1 ps [36]. The 50 ns simulations were used for data analysis.

### 2.3. Ligand Binding Energy

The Molecular Mechanics Poisson–Boltzmann surface area (MM-PBSA) method [37] was used to calculate the binding energy of each ligand to the protofibril. This method is based on the single-trajectory approach. Thus, 100 snapshots collected consecutively over the course of the 50 ns simulations, once the ligand reached a stable binding (i.e., Root Mean Square Displacement of its center of mass <5 Å; Figure S1), were used. The binding free energy (ΔG_binding_) is described as the free energy difference between the complex, G_complex_, and the summation of the free energy of the protein, G_protein_, and ligand, G_ligand_:

(1) $$\Delta G_{\mathrm{binding}}=G_{\mathrm{complex}}-\left(G_{\mathrm{protein}}-G_{\mathrm{ligand}}\right)$$

The free energy of each molecule is given by

(2) $$G=E_{\mathrm{MM}}+G_{\mathrm{solvation}}-T\Delta S$$

where T and S represent the temperature and entropy, respectively; and the mechanical energy, E_MM_, of the solute in the gas phase is given by the summation of bond, angles, dihedrals, Van der Waals, and electrostatic terms:

(3) $$E_{\mathrm{MM}}=E_{\mathrm{bond}}+E_{\mathrm{angle}}+E_{\mathrm{dihedral}}+E_{\mathrm{electr}}+E_{\mathrm{VdW}}$$

The solvation energy, G_solvation_, is calculated as follows:

(4) $$G_{\mathrm{solvation}}=G_{\mathrm{surf}}+G_{\mathrm{PB}}$$

where the nonpolar solvation term, G_surf_, is approximated on the solvent-accessible-surface area (SASA) derived from the Shrake–Rupley numerical method [38]:

(5) $$G_{\mathrm{surf}}=\gamma \mathrm{SASA}+\beta$$

with γ = 0.0072 kcal/mol Å^2^ and β = 0 [39].

The term comprising the electrostatic potential between the solute and the solvent, G_PB_, is calculated using the continuum solvent approximation [40] by the APBS package [41].

The entropy term, *T*Δ*S*, is computed using the quasi-harmonic formula [42].

### 2.4. Aβ(1–40) Oligomer Double-Layered Structures

**Figure 1:**
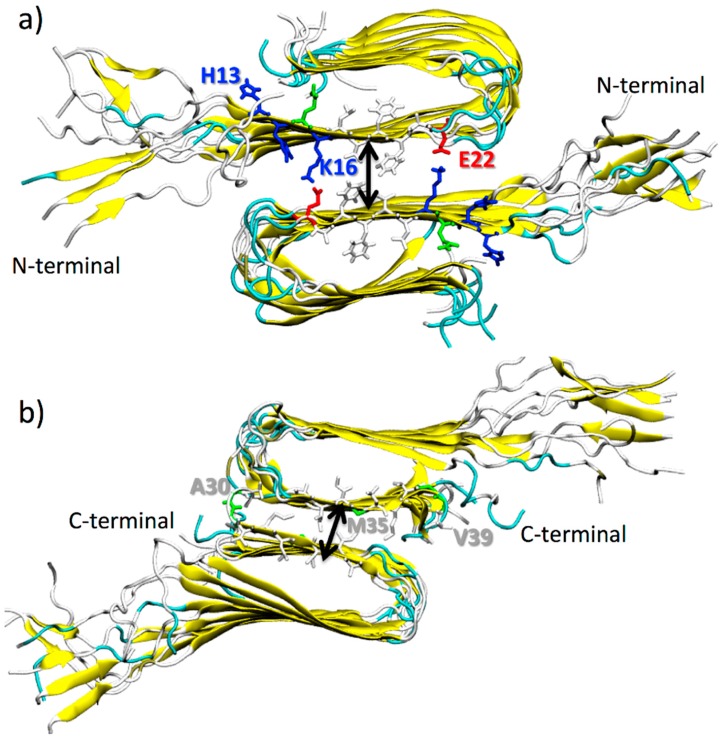
Cartoon representation of double-layered structures of Aβ(1–40) oligomers facing through their β-1, in (**a**), and β-2 β-sheets, in (**b**). Fibrils are colored according to their secondary structures. Amino acids at the interface are explicitly represented (color code: blue for positively charged, red for negatively charged, and white for hydrophobic amino acid residues). Black arrows roughly represent the intersheet distance.

Two possible double-layered structures were built by stacking the β-sheets of each monomer onto each other in an antiparallel fashion [43,44], as shown in Figure 1. The C-terminal–C-terminal and N-terminal–N-terminal interfaces were thus obtained. The intersheet distance was computed as the distance between the centers of mass of the two β-sheets that are in contact. The amino acids that were considered for the calculations of the center of mass are H13, H14, Q15, K16, L17, V18, F19, F20, A21, and E22 for the N-terminal–N-terminal interface (β-1 β-sheets) (Figure 1a) and A30, I31, I32, G33, L34, M35, V36, G37, G38, and V39 for the C-terminal–C-terminal interface (β-2 β-sheets) (Figure 1b).

In order to evaluate the influence of the ligands on the stability of the different protofibrils polymorphs, the potential of mean force (PMF) method implemented in the GROMACS program was used [45,46].

**Figure 2:**
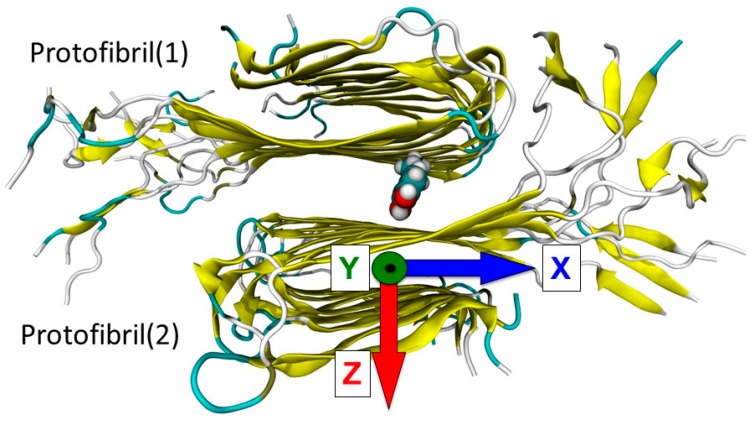
Pulling directions applied to protofibril (2) during the calculation of the forces needed for double-layered destruction: along the x-axis (i.e., outward shift of the protofibril (2) along its secondary axes), the y-axis (i.e., lateral shift of the protofibril (2) along its primary axes), and the z-axis (i.e., vertical shift, progressive removal of protofibril (2)).

The backbone of protofibril (1) was restrained in its starting position, while a force increasing with time was assigned to the center of mass of protofibril (2). Three directions were taken into account, as shown in Figure 2: the x-axis (i.e., outward), the y-axis (i.e., lateral), and the z-axis (i.e., vertical). For each ligand and for both protofibril contact modes (β-1 and β-2 β-sheets), three runs were performed, using as the starting configurations the ones at 90, 95, and 100 ns, ensuring good sampling. The starting force used at the beginning of the simulation was 1000 kJ/mol nm^2^, and the rate at which the application point of the force moves was 0.01 nm/ps.

## 3. Results and Discussion

The five natural compounds studied are listed in Table 1, together with their effective concentrations (EC_50_) for the formation, extension, and destabilization of preformed Aβ(1–40) (fAβ(1–40)).

The overall in vitro activities of curcumin (CUR) and rosmarinic acid (ROSM) are similar [47]. Moreover, in vivo observations suggest that curcumin may be beneficial even after the disease has developed, reducing the amyloid levels and plaque burden of aged mice with advanced amyloid accumulation [48]. Quercetin (QUER) shows moderate in vitro preformed fAβ(1–40) destabilization effects with respect to CUR [49]. (-)-Epigallocatechin-3-gallate (EGCG) is undergoing phase II–III clinical trials as an inhibitor of Aβ fibrillogenesis. It decreases plaque burdens in the brain and reduces soluble and insoluble preformed fAβ(1–40)s [50]. Finally, dopamine (DOPA) proved to be a potent anti-amyloidogenic agent at all the different levels of formation, extension of amyloid fibrils, and destabilization of preformed fAβ(1–40)s [51].

Heterogeneity in the experimental conditions (i.e., peptide concentrations, incubation condition, and procedure of fAβ preparation) used in different laboratories or different experiments in the same laboratory gives rise to discrepancies in effective EC_50_ concentrations, thus preventing a quantitative rationalization of the observed experimental trend by means of the results of the computational simulations. However, some interesting qualitative structure–activity relationships could be considered, as shown in the following.

### 3.1. Putative Binding Sites and Binding Free Energies

Six main binding sites have been highlighted by means of the REMD method applied to the ligands considered. They are located at the surface of the protofibril:

1. β-1 β-sheet corresponding to the amino-acid sequence: ^16^KLVFFAEDV^24^,
2. β-2 β-sheet corresponding to the amino-acid sequence: ^31^IIGLMVG^37^,
3. Elbow connecting the two β-sheets with the corresponding amino-acid sequence: ^22^EDVGSN^27^,
4. top of the protofibril, over the two β-sheets of the terminal Aβ(1–40) monomer (“Over”),
5. disordered tails located at the N-terminal,
6. end of the β-2 β-sheet, on the C-terminal (entry of the cleft).

**Figure 3:**
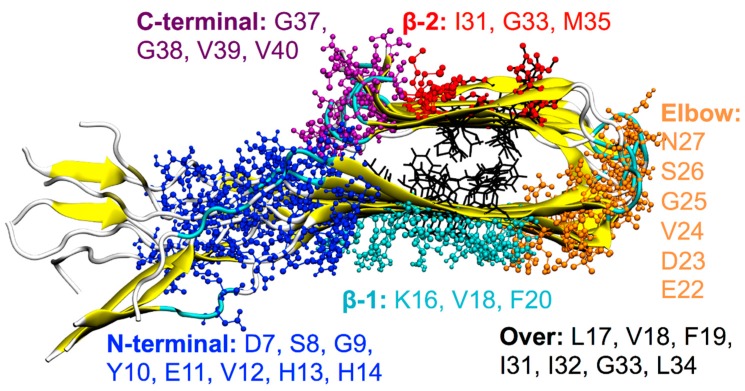
Ball-and-stick representation of the ligand binding sites obtained by REMD. Amino acids (single-letter code) involved in the interactions are reported for each binding site with different colors: Amino acids belonging to the N-terminal site are in blue, to the the β-1 site in cyan, to the Elbow site in orange, to the β-2 site in red, to the C-terminal site in purple, and to the Over site in black).

**Figure 4:**
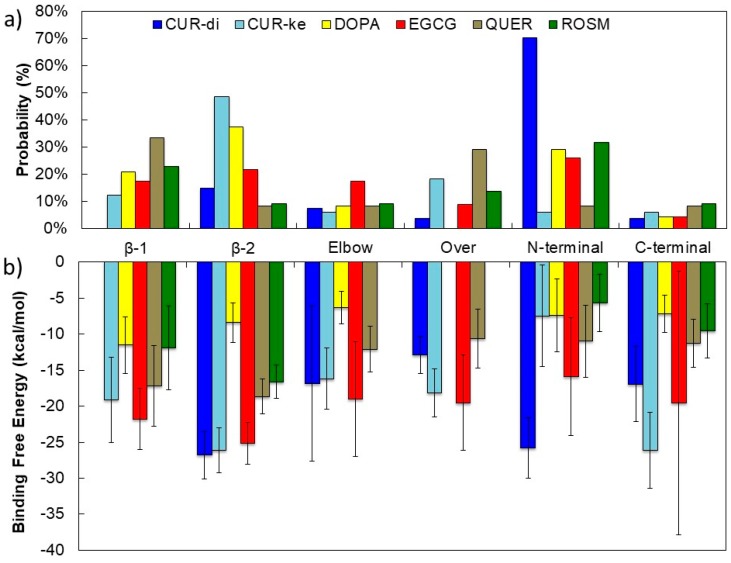
Probability of occupancy of each binding site (**a**) and binding free energy (**b**) for each ligand considered.

For each binding site, amino acids that make persistent interactions (in this work, an interaction is considered as persistent if the amino acid residue remains in contact with the ligand for at least 60% of the total simulation time) with the ligands and that contribute more than 1 kcal/mol to the binding energy are highlighted in Figure 3. The probability of the occupancy of each site is shown in Figure 4a.

It is interesting to note the different occupancy preferences of the two forms of curcumin. The CUR-di form predominantly interacts with the N-terminal, whereas CUR-ke is mainly found at the β-2 site.

Multiple binding sites have been previously described in the literature for curcumin derivatives and other related compounds. In particular, the β-2 site has been very recently targeted in a combined computational and experimental study by Battisti et al. [15], aimed at the design of curcumin-like amyloid beta peptide inhibitors. Binding to the N-terminal and Over positions have been observed for curcumin and other ligands by means of site map analysis by Kundaikar et al. [52]. Moreover, the β-1 binding site has previously been suggested as a possible binding site for curcumin on the basis of solid-state NMR experiments [53] and computational studies on the Aβ hexapeptide ^16^KLVFFA^21^ and full-length Aβ fibrils [12,15].

Although a few studies in the literature proposed the cavity formed by the two β-sheets and the turn as a possible binding site for curcumin [17,18] and other compounds such as Orange-G [19], this site is never occupied by the ligands considered in the present study. However, small portions of the CUR-ke, EGCG, QUER, and ROSM ligands can occasionally penetrate this cavity during the dynamic simulations runs, when they are interacting with the Aβ(1–40) protofibril in the Over position.

By considering the probability of the occupancy of each binding sites (Figure 4a) together with the corresponding binding free energies (Figure 4b), it emerges that:

- CUR-ke, the predominant form in aqueous solution on the basis of the recent results obtained by Manolova et al. [54], shows a strong propensity to dock at the β-2 site and realizes at this site strong interactions (ΔG_bind_ > −20 kcal/mol) with the fAβ(1–40) fibril. However, moderate to strong (−10 < ΔG_bind_ > −20 kcal/mol) free energies of binding are found for all the binding sites, with the exception of the N-terminal (N-ter) one.
- DOPA shows a preference for docking at the β-2 and N-ter sites. However, by considering the free energy of binding, it does not show selectivity among the six sites studied, realizing moderate to weak interactions (G_bind_ < −10 kcal/mol) with all of them.
- EGCG preferentially targets the N-ter and β-2 sites and secondarily, the Elbow and β-1 sites. However, this ligand is able to realize strong binding with all six possible sites. The most stable complexes (ΔG_bind_ > −20 kcal/mol) are obtained at the β-2 and β-1 sites. The ability of EGCG to bind to the N-terminal amino acids (residues 1–16) is confirmed by results obtained by isothermal titration calorimetry experiments [55]. Moreover, recent findings by solution NMR indicate that EGCG preferentially binds to Aβ oligomers and shields them at the β-1 and β-2 sites [56], where it remodels the oligomer surface, altering the interactions with the monomers.
- QUER is found almost equally distributed between the β-1 and Over sites, with significantly lower probability for the other sites. However, it realizes moderate binding free energies (ΔG_bind_~−10 kcal/mol) in all sites, with the most stable complexes (ΔG_bind_~−20 kcal/mol) involving the β-2 and β-1 sites. These results are in agreement with the finding of a computational study recently reported by Ren et al. [13] for a structurally homologous compound, genistein. They showed that genistein prefers to bind the β-sheet grooves to interfere with their self-aggregation.
- ROSM has higher probability for docking at the N-ter and β-1 sites, but realizes the most stable interactions with moderate binding free energies at the β-2 and β-1 sites. Indeed, NMR investigations suggest that a ROSM hairpin-like structure would allow the intercalation into the Aβ oligomers structure at the interprotofilament (β−β zippers) interface [57].

Thus, taking the error in the computation of the ΔG_bind_ into account, it can be stated that the β-2 groove is a common structural target for all the ligands studied; at this site, the ligands realize their most stable interactions with residues M35, G33, and I31. The β-1 site is also targeted for energetically favored complexes, realized mainly by the interaction with the K16, V18, and F20 residues.

These regions are particularly interesting since they constitute the junction between protofilaments in common Aβ(1–40) polymorphs [24,58]. Several recent computational studies employing different multiple protofilament structures and a variety of ligands, used as markers for amyloid detection, indicate the interfacial pockets at the junction between protofilaments as preferential binding sites [19,20,21]. Binding of ligands at these sites can interfere with the formation or induce the disruption of the aggregates, as discussed in the next section.

In agreement with the previous studies on related compounds [9,47], the binding free energies obtained for these complexes are driven by more favorable nonpolar interactions rather than by electrostatic ones (Figure S2).

**Figure 5:**
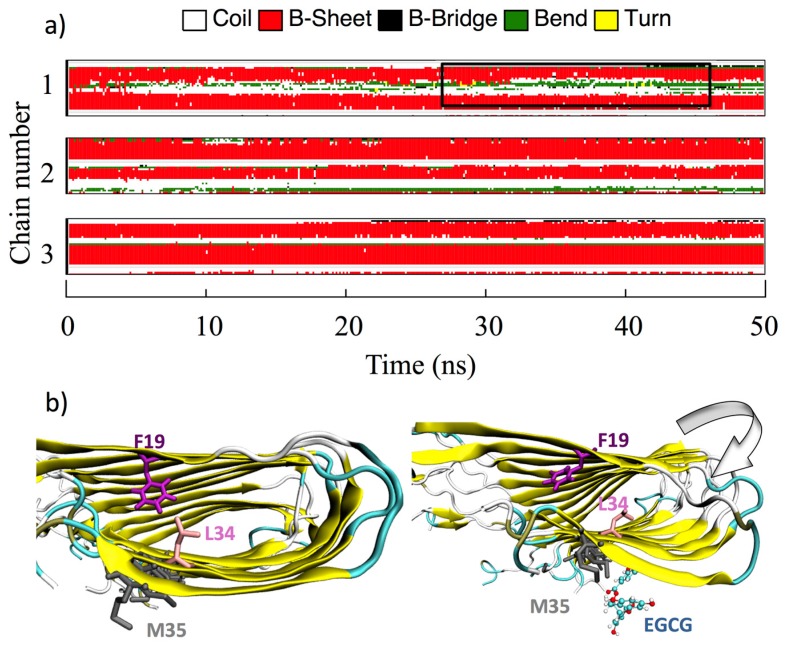
(**a**) Time evolution of the Aβ(1–40) secondary structure (computed with the GROMACS DSSP tool) upon EGCG binding on the β-2 β-sheet groove. The perturbation induced at the monomers lying at the head of the protofibril is highlighted by a black box. For clarity’s sake, only the top three Aβ(1–40) monomers are shown. (**b**) Conformation of F19 and L34 before (left) and after (right) the interaction of EGCG with M35.

Visual inspection of all MD trajectories shows that the random-coil N-terminal ^1^DAEFRHDS^8^ sequence does not appreciably alter the conformation and the usual behavior of the rest of the fibril, despite its high flexibility, promoting the nomadism of the ligands that bind preferentially to D7 and S8. Moreover, overall, the binding of the ligands does not disturb the structural integrity of the Aβ protofibrils, their overall U-shaped conformations being retained with or without interacting ligands. The secondary structure of the Aβ monomers forming the core of the protofibrils remains unperturbed upon ligand binding in the time length of the simulations, whereas β-sheet unfolding is observed for the first two monomers at the top and bottom of the protofibril. This is shown in Figure 5, where the time evolution of the Aβ(1–40) secondary structure upon EGCG binding on the β-2 β-sheet groove is reported: chain 3 is representative of the core monomers from 3 to 8 in the simulated protofibril, whereas chains 1 and 2 are representative of the top two (bottom two) monomers.

However, in a few cases (EGCG, CUR-ke, and QUER) when the ligands, during the dynamic run, migrate from the Over site to the β-2 β-sheet in proximity to M35, a perturbation of the fibril secondary structure in the terminal monomers lying at the head of the protofibril is observed. This perturbation, observed in the time of the simulations, especially in the elbow region, induces a bend in the long fibril axis that can impair the process of fibril elongation.

Figure 5 explains the phenomenon for the complex formed by EGCG and the Aβ protofibrils. The side chain of M35, interacting with the ligand, chaperones it in the search for the best interactions in the β-2 groove, causing a bending of the protofibril and altering the Aβ protofibril secondary structure in the Elbow region. Moreover, the dynamics of the M35 side chain, induced by the interacting ligand, disrupts the hydrophobic interaction between L34 and F19, which is found to influence a broad range of different processes including the initiation of fibrillation, oligomer stability, fibril elongation, and cellular toxicity [59]. In addition, it is worth underlining that M35 itself is also known to be responsible for the hierarchical assembly of amyloid fibrils.

### 3.2. Influence of the Ligands on the Stability of the Aβ(1–40) Oligomer Double-Layered Structures

The effect of the ligands on the stability of the protofibril double-layered structures has been quantified by the calculation of the forces (PMF) for protofibril(1)/ligand–protofibril(2) unbinding. On the basis of the binding site preferences discussed in the previous section, the intercalation of the ligands into the C-terminal–C-terminal and N-terminal–N-terminal interfaces of the protofibrils have been considered. Moreover, three possible ways for complex disruption have been examined by applying the forces along the x-axis (i.e., outward shift of the protofibril(2) along its secondary axes), the y-axis (i.e., lateral shift of the protofibril(2) along its primary axes), and the z-axis (i.e., vertical shift, progressive removal of protofibril(2)), as shown in Figure 2.

The results are reported in Table 2, together with the force needed to separate the pristine protofibril–protofibril aggregation, taken as the control.

It is worth noting that the C-terminal–C-terminal interface of the double-layered Aβ-sheets consists of highly hydrophobic patches of I31, I41, and M35, with an average intermolecular distance between the two β-sheets of ~9.1 Å (see Table 3), whereas the N-terminal–N-terminal interface consists of both hydrophobic patches of V18 and F20 and K16–E22 salt bridges, with an average intermolecular distance of ~14.3 Å, in agreement with previous computational studies on Aβ17–42 [60] and on different segmental polymorphs (Aβ 35–42, Aβ 16–21, Aβ 27–32) modelled by Berhanu et al. [61]. These characteristics determine the stability of the β-sheet–β-sheet interfaces, which is significantly higher for the N-terminal–N-terminal arrangement with respect to the C-terminal–C-terminal one, as indicated by results from the PMF for protofibril(1)–protofibril(2) unbinding, at least for the vertical and outward directions (Table 2).

Overall, the binding of the ligands to β-sheet–β-sheet interfacial pockets located between two protofilaments produces a reduction of the stability of the protofibril dimeric structures. However, this cannot be directly correlated to the increasing in the intermolecular distances between the two interacting protofibrils. In fact, for the N-terminal–N-terminal interface, the distance increase upon ligand binding is in the order of 2 Å, while for the C-terminal–C-terminal one, initially characterized by a tight binding due to hydrophobic interactions, it is ~4–5 Å (Table 3).

On the other hand, the maximum destabilization of the double-layered Aβ-sheet aggregates is observed for the β1-arrangements, when the forces are applied along the vertical (y), outward (z), and lateral (x) axes, in that (descending) order.

The binding of ligands at the C-terminal–C-terminal interface results in a moderate destabilization of the double-layered Aβ-sheet aggregates with respect to the lateral and vertical modalities, whereas for the outward disruption, it appears that the ligands have no effect or confer a small stabilization of the complexes; the large errors obtained do not allow further lucubration.

It is worth noting that the intersheet separation produced by DOPA, the smallest ligand, is larger or comparable to the one observed for more cumbersome ligands, and its effect on the destabilization of the protofibril dimeric aggregates is also overall stronger than the other ligands.

## 4. Concluding Remarks

The results of the systematic computational study carried out on the interaction of five natural biophenols with single Aβ(1–40) protofilaments by means of REMD simulations allowed the individuation of multiple binding sites for each ligand, located at the surface of the protofibril near to the β-1 β-sheet, β-2 β-sheet, elbow connecting the two β-sheets, top of the protofibril, disordered N-terminal, and the C-terminal.

The REMD methodology used does not allow the biophenols to enter into the hydrophobic core of the preformed protofibril, probably because the energy penalty associated with the penetration process cannot be overcome using conventional MD. The absence of binding sites in the cavity of the preformed protofibril prevents the study of destabilizing effects of the ligands by promotion of disruption of the native backbone hydrogen bonds in the protofibril interior.

The MM-PBSA energetic analysis of the binding shows that the β-1 and β-2 binding sites at the exposed surface of the Aβ(1–40) protofibrils, shared by all the five ligands studied, are thermodynamically favored. At these sites, the anti-amyloid activity of biophenols consists in the inhibition of fibril thickening and elongation.

In fact, although no significant perturbation of the overall protofibril secondary structure is observed in the periods of time studied, interesting conformational changes of the terminal peptides with subsequent bending of the principal axis of the protofibril are induced by ligands that migrate during the dynamic run from the Over binding site to the β-2 binding site. This effect is more marked for EGCG, but is observed also for CUR-ke and QUER and may preclude the association of an incoming Aβ peptide inhibiting the fibril elongation.

Moreover, ligand binding at the β-2 binding site may inhibit the amyloidogenic process by shielding the M35, which is responsible for the hierarchical assembly of amyloid fibrils, and disrupting the hydrophobic interaction between L34 and F19, which is found to influence a broad range of different processes including the initiation of fibrillation, oligomer stability, fibril elongation, and cellular toxicity.

Finally, the stability of the β-sheet–β-sheet interfaces of the Aβ(1–40) oligomer double-layered structures is significantly affected by the intercalation of the biophenols. The force needed for disruption of the aggregates is halved by all the ligands binding the N-terminal–N-terminal interface when the forces are applied along the principal axis of the protofibril. The most remarkable effect is observed for DOPA on the double-layered structure in the N-terminal–N-terminal arrangement, whatever the force direction; whereas ROSM and EGCG exert a stronger destabilization at the double-layered structure in the C-terminal–C-terminal arrangement.

These structural insights may serve as a molecular guide for setting up further rational drug design in close collaboration with experimentalists in order to obtain effective inhibitors targeting fibril formation in Alzheimer’s disease.

## Figures and Tables

**Table 1:** The effective concentrations (EC_50_) of the ligands studied for the formation, extension, and destabilization of fAβ(1–40).

Compound	Acronym	Structure	Aβ(1–40) Formation (EC_50_) μM	Aβ(1–40) Extension (EC_50_) μM	Aβ(1–40) Destabilization (EC_50_) μM
Curcumin diketo form	CUR-di	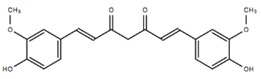	0.19 [47]	0.19 [47]	0.42 [47]
Curcumin ketoenol form	CUR-ke	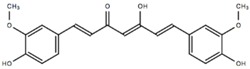	0.81 [48]	0.19 [47]	1.00 [48]
Dopamine	DOPA	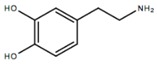	0.01 [51]	0.03 [51]	0.21 [51]
(-)-Epigallocatechin-3-gallate	EGCG	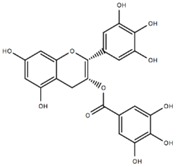	0.18 [4]	-	15 * [50]
Quercetin	QUER	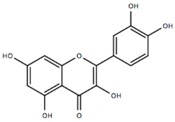	0.24 [49]	0.25 [49]	2.1 [49]
Rosmarinic acid	ROSM	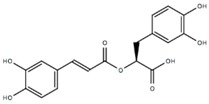	0.29 [47]	0.26 [47]	0.83 [47]

* Referred to Aβ(1–42) fibrils.

**Table 2:** Computed force (expressed in kJ/mol) needed for protofibril(1)–protofibril(2) (control) and protofibril(1)/ligand–protofibril(2) unbinding along the x, y, and z-axes.

Force Direction	Lateral (x-axis)		Vertical (y-axis)		Outward (z-axis)	
Ligand/binding site	β-1	β-2	β-1	β-2	β-1	β-2
Control	2743 ± 115	2772 ± 140	3520 ± 200	2013 ± 30	3413 ± 250	2387 ± 330
CUR-di	2573 ± 40	1913 ± 110	1570 ± 70	1843 ± 35	2810 ± 10	2107 ± 140
CUR-ke	2600 ± 100	2167 ± 280	1653 ± 60	1733 ± 150	2760 ± 70	2633 ± 250
DOPA	2356 ± 95	2180 ± 190	1663 ± 55	1927 ± 420	2150 ± 95	2540 ± 90
EGCG	2968 ± 93	2407 ± 75	1967 ± 25	1610 ± 115	2570 ± 30	2533 ± 60
QUER	2493 ± 90	2043 ± 155	1726 ± 75	1720 ± 30	2553 ± 120	2650 ± 100
ROSM	2888 ± 173	1677 ± 55	2053 ± 40	1367 ± 15	2767 ± 70	2310 ± 105

**Table 3:** Intersheet distance in the Aβ(1–40) oligomer double-layered structures.

	β-1	β-2
Control	14.3 ± 0.3	9.1 ± 0.3
CUR-di	15.7 ± 0.4	13.4 ± 0.3
CUR-ke	15.4 ± 0.3	13.6 ± 0.4
DOPA	16.5 ± 0.5	14.1 ± 0.4
EGCG	16.6 ± 0.5	13.0 ± 0.4
QUER	16.3 ± 0.4	12.7 ± 0.4
ROSM	15.8 ± 0.3	14.1 ± 0.3

## Supplementary Materials

The following are available online. Figure S1: Root Mean Square Deviation (RMSD) of the center of mass of EGCG during the simulation time; Figure S2: Decomposition of free energy contributions in the MM-PBSA calculation for each ligand tested. ΔE_VdW_ in red, ΔE_elec_ in green, ΔE_PB_ in purple, ΔE_SASA_ in cyan, Entropy in blue.
