# Supplementary material for: Computational Insight into the Effect of Natural Compounds on the Destabilization of Preformed Amyloid-β(1–40) Fibrils
Source: Molecules. 2018 May 31;23(6):1320. doi: 10.3390/molecules23061320 (PMC6100107; doi:10.3390/molecules23061320)
Supplement: Supplementary file 1 [file molecules-23-01320-s001.pdf]

Supplementary Materials

Computational insight into the effect of natural compounds on the assembly of amyloid-β(1-40) fibrils

Francesco Tavanti,* Alfonso Pedone and Maria Cristina Menziani*

Department of Chemical and Geological Sciences, University of Modena and Reggio Emilia, Via G. Campi, 103 – Modena, Italy.

*To whom correspondence should be addressed
E-mail: mariacristina.menziani@unimore.it, francesco.tavanti@unimore.it

Figure S1: Root Mean Square Deviation (RMSD) of the center of mass of EGCG during the simulation time.

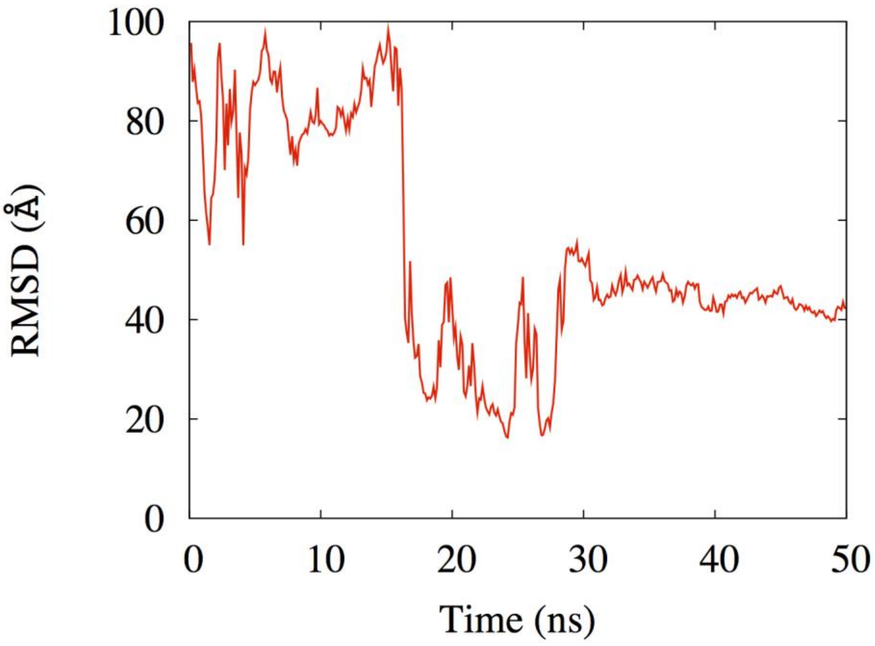

Figure S2: Decomposition of free energy contributions in the MM-PBSA calculation for each ligand tested. ΔE_{VdW} in red, ΔE_{elec} in green, ΔE_{PB} in purple, ΔE_{SASA} in cyan Entropy in blue.

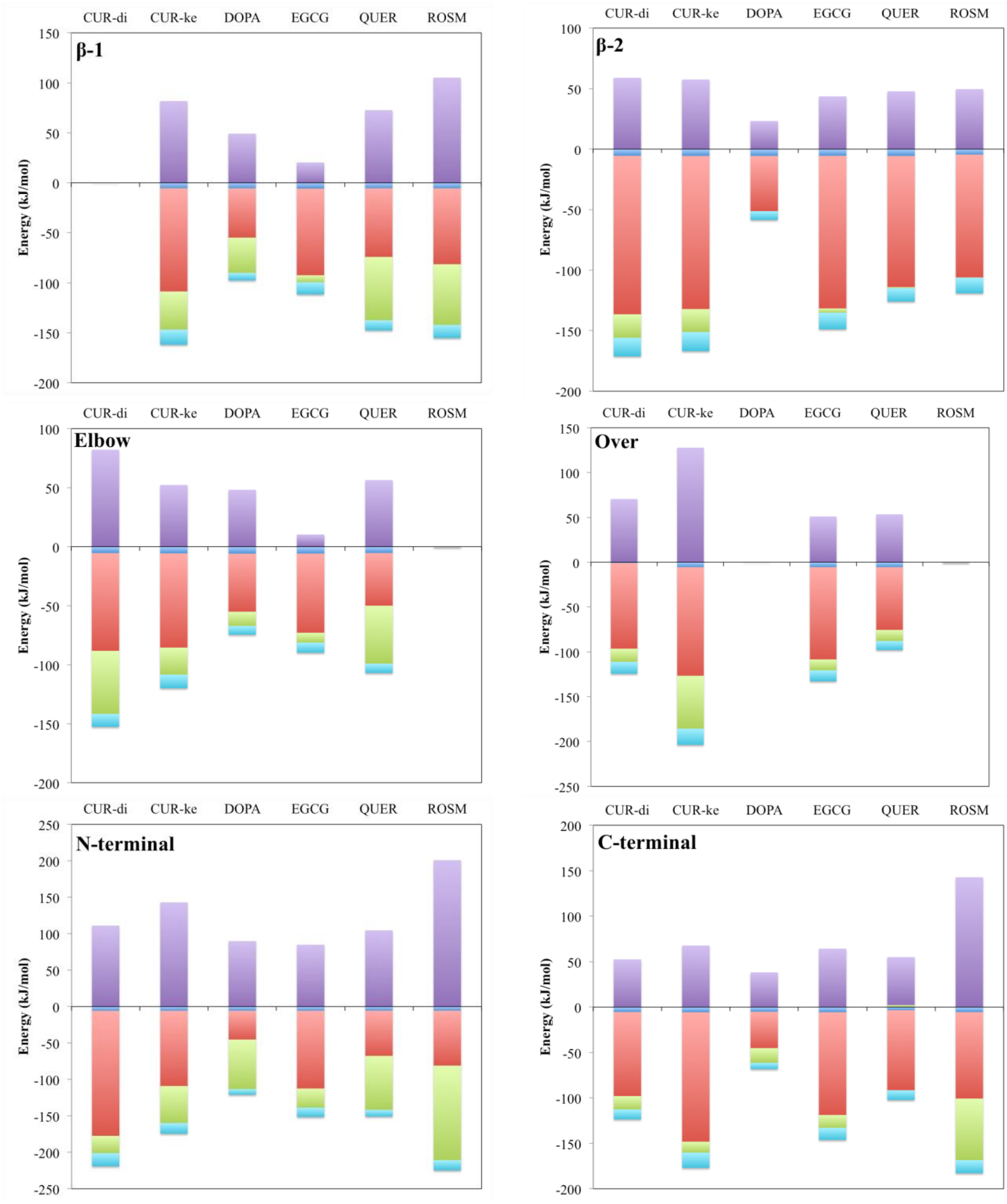

$$\Delta G_{binding} = \Delta G_{VdW} + \Delta G_{elec} + \Delta G_{solv} - T\Delta S$$

$$\Delta G_{solv} = \Delta G_{PS} + \Delta G_{SASA}$$

Where ΔE_{elec} is the non-solvent electrostatic potential energy; ΔG_{PB} the electrostatic contributions to the solvation free energy calculated with Poisson-Boltzmann equation; ΔG_{SA} the non-polar contributions to solvation free energy; ΔE_{vdw} the van der Waals potential energy; and $\Delta G_{binding}$ the calculated binding. ΔG_{solv} are the polar, non-polar and total solvation energies.

Electrostatic and van der Waals terms in the gas phase provide the major favourable contributions to the polyphenols binding, whereas the polar contribution to the solvation energies impair the binding.
